# Supplementary material for: Determinants of COVID-19 Vaccination Intention among Health Care Workers in France: A Qualitative Study
Source: Vaccines (Basel). 2022 Oct 4;10(10):1661. doi: 10.3390/vaccines10101661 (PMC9611955; doi:10.3390/vaccines10101661)
Supplement: Supplementary file 1 [file vaccines-10-01661-s001.zip › vaccines-1923979-supplementary.pdf]

vaccines

MDPI

Table S1. Analysis grid of semi-structured interviews carried out among health care professionals at the Bordeaux university hospital, from January to April 2021, France

N°	Categories	Subcategories
1	Experience effect	1. Personal experiences (itself, family, friends, colleagues) with drugs or vaccines<br>2. COVID-19 experiences
2	Relationship to care	1. Vaccine habits (ex. flu)<br>2. Lives habits (limit care, medications, use of alternatives medicines)<br>3. Opinion about vaccination in general
3	Relationship to COVID-19 vaccines proposed	1. Fear of side effects<br>2. Level of confidence in COVID-19 vaccines available<br>3. Preference for a specific vaccine (laboratory, technology, origin)<br>4. Level of satisfaction according to vaccines modalities (efficiency, used technologies, protection period)
4	Posture adopted in relation to vaccination<br>(how HCW is positioned in relation to the decision to vaccinate)	1. Need for time to reflect (additional scientific data, observation of effects on the first vaccinated)<br>2. Posture considered: feeling of not being concerned by vaccination, expectation of being concerned/priority public, feeling of being a priority<br>3. Sense of obligation to vaccinate
5	Advantages perceived of COVID-19 vaccination	1. To return to a normal life (professional and personal)<br>2. To protect the most vulnerable and the patients<br>3. Collective immunization<br>4. To protect their family and friends<br>5. Individual protection<br>6. To curb the epidemic, limit the spread
6	Benefits / risks of COVID-19 vaccination	1. Perception of vulnerability (exposure to disease, specific pathologies, etc.)<br>2. Perception of the severity of COVID-19 disease<br>3. Perception of the effectiveness of barrier measures<br>4. Perception of the effectiveness of the vaccine for oneself, spread of the epidemic
7	Social norms	1. Interaction with surroundings (personal and professional)<br>2. Taking into account of opinion of vaccine status of the environment (surroundings, others countries)<br>3. Posture taken (attitude/recommendations)<br>a. Colleagues at work<br>b. Patients<br>c. Surroundings (parents, friends, partners, etc.)

8	Level of confidence	1. In political and health authorities<br>2. In health care workers, experts in health, immunization<br>3. Media<br>4. Hierarchy<br>5. Personal valorization, confidence perceived by the interviewed
9	Relationship to scientific information and media	1. Level of knowledges perceives<br>2. Feelings about information in the health context (media, personal, professional)<br>3. Access to information at work<br>4. Expectations expressed (stability of information, de-dramatization and objectivity of the media)
10	Relationship to vaccine campaign	1. Role/involvement expressed in the campaign<br>2. Opinions about the organization<br>3. Desired organizational elements (University Hospital Center, national level)<br>4. Organizational challenges identified<br>5. Level of knowledge about the vaccination campaign

Table S2. Qualitative themes and exemplar quotes

Theme	Exemplar quotes
	Low level of trust
Confidence in the vaccines being offered	« We don't have enough perspective and it's true that it scares us » *(A1, healthcare assistant)*<br>« I think the COVID-19 vaccine roll-out has been really fast (...) you get the impression that the elderly are being used as guinea pigs » *(A1, healthcare assistant)*<br>« The disease is so recent (...) and the vaccine was made very quickly. When we know, for example, that the vaccine against AIDS is still not available, since the time we have been searching...» *(A1, healthcare assistant)*<br>« I am still a little puzzled about the effectiveness of a vaccine that was given so quickly (...) I don't want there to be any consequences following the vaccination » *(A4, healthcare assistant)*<br>« It's something that was done in 2 times 3 movements. There is no hindsight, there is nothing » *(B3, nurse)*<br>« It's a vaccine that is still young, we still don't have much information about the side effects » *(D1, hospital officer)*<br>« We have a little questioning with this RNA new method » *(F1, porter)*<br>« The fact that it's fast (…) we are still suspicious (…) we don't really know what is at stake (…) if it is really to protect people or to make money» *(G2, physiotherapist)*<br>« So vaccination, uh, I must admit that I'm a little worried because it happened so suddenly that... It's true that I'm worried (...) It's frightening to think that somewhere, in order to win in the short term, they're putting people's health at risk in the more or less long term » *(E1, administrative staff)*<br>« If in 2/3 years they find something that doesn't have RNA in it, why not. But as long as it's RNA it's a no go » *(B3, nurse)*
	High level of trust
	« There were no questions on my part about the side effects, since that's what a vaccine is all about (...) I had no fears» *(C4, physician)*<br>« I am rather confident about the interest of the vaccine though» *(B4, nurse)*<br>« I know the studies that say that we still have no assurance of transmission. Now I think that a vaccine that works on the virus and on the severe form necessarily decreases the viremia, so it necessarily decreases the risk of transmission. I am quite convinced of the subject » *(C2, physician)*<br>« I think that we have to live with the times and that at the moment, the only evidence we have is that vaccines work. And then we'll see in 200 years» *(C3, emergency physician)*
	With vaccination
Experience effect	« Me, in addition, in the family there have already been problems with the vaccination » *(A1, healthcare assistant)*<br>« Twice in a row I got a flu vaccination... I was sick! More than when I normally get the flu (...) I am against the flu shot, so I am against the covid» *(A2, healthcare assistant)*

	« The flu, I admit I didn't get it. It's the only one that scares me. Vaccinations, let's say... basic, I do them, but the flu, no » *(A5, healthcare assistant)*<br>« I never had a flu shot (...) I have a cousin who had never had a flu shot, she had a fever of 40. She was afraid for a week, she was in bed, aching (...) so since then, no way» *(A6, healthcare assistant)*<br>« It's out of the question [to be vaccinated], it's like hepatitis B. There will be multiple sclerosis behind it, what will happen? » *(B3, nurse)*<br>« I'm not someone who vaccines himself against the flu for example (...) I did it only once because I had boxing competitions and the night after I was sick » *(F1, porter)*<br>« I have a friend who was vaccinated against hepatitis B (...) and who ended up with multiple sclerosis, for example » *(E1, administrative staff)*<br>« For me, having lived through a good flu episode, I don't want to go through that again » *(H1, senior nurse)*<br>« I think that our generations are lucky, we don't see people limping because of polio (...) People forget » *(C1, physician)*<br>« I've had the flu shot every year since I was 17, since I had the flu » *(C3, emergency physician)*<br><br>*With COVID-19 disease*<br><br>« It's been a year that I've been very careful, that my life has been completely turned upside down (...) I don't have the possibility of staying at home so as not to expose myself, I was obliged to go there... With people who were contagious and we knew it » *(A3, healthcare assistant)*<br>« I've lost old people in my family in the last year (...) I think you see things differently when you're in it (...) I got COVID-19. I saw people die one by one, it's really scary » *(A5, healthcare assistant)*<br>« I don't want to have it again because I still have after-effects » *(B2, volunteer nurse at the vaccination center)*<br>« I'm in the emergency room, I'm confronted every day with patients who come in, I think we're all sick of it » *(C2, physician)*<br>« I think I've been exposed to it, now I'm not really afraid of getting the virus. I think I've either had it already or I'm immune » *(B4, nurse)*
The opinions and immunization status of others	« When they [her colleagues at work] have been vaccinated, they will tell us how they felt after-wards and how they feel now, and that can reassure us too » *(A1, healthcare assistant)*<br>« The fact that in the whole world there are not so many cases (...) that have had big side effects, that's what is very reassuring » *(A3, healthcare assistant)*<br>« For my close circle of friends, I think it can reassure them [that she is vaccinated]. As for me, I think it can reassure me too » *(A5, healthcare assistant)*

	« The day they tell me "*well 9 out of 10 people are vaccinated, and uh we're almost there, well go ahead*" well then I'll think about it a little more » *(B1, nurse)*<br>« We have this feeling of having done everything right, and the fact of doing well is always rewarding» *(C3, emergency physician)*<br>« Well, I'll play it communal (...) Vaccines have always worked by vaccinating as many people as possible (...) If it's not successful, I won't get vaccinated» *(G1 , physiotherapist)*<br>« If only a small percentage of the population was vaccinated, well, I wouldn't go running to be vaccinated first » *(G1, physiotherapist)*<br>« If nobody does it... what's the point? (...) The thing that could possibly make me change my mind is if I realize that there is only one nurse in my department who is vaccinated... Then I will ask myself whether I should be vaccinated » *(B4, nurse)*<br>« I think that by discussing it, we can reach a consensus, and say to ourselves "*yes, it may be worthwhile to be vaccinated*"» *(E1, administrative staff)*
Media influence on public opinion	« I don't think I will do it [COVID-19 vaccination] to begin with. Because we hear so much about anything and everything ... I would prefer to wait a little and not rush » (*A4, healthcare assistant*)<br>«To hear on TV that it would be potentially less effective than another [AstraZeneca vaccine], I don't find that reassuring at all» *(A4, healthcare assistant)*<br>« It is also necessary to listen elsewhere what, not to focus on the angle of view of the media which want to make buzz » *(C1, physician)*<br>« Here, the communication on AstraZeneca vaccine was not extraordinary I find» *(C2, physician)*<br>« We are always alarmist about everything. (...) You never get the impression that you are doing things right, no matter what you do. The count of the dead, after the count of the contaminated. But are any of them really serious? (...) It's the media hype. In all of this, a little bit of positivity, yeah, it wouldn't hurt » *(G2, physiotherapist)*<br>« The media is playing on the negative card to scare people, to put some pressure on people to go and get vaccinated » *(G2 physiotherapist)*<br>« What we can read today in the press, I do not really know if we can take for granted » *(G1, physiotherapist)*<br>« It's the current atmosphere in fact... We hear everything and its opposite... And it's true that it leaves an uncertainty in our minds » *(F1, porter)*<br>« I prefer scientific studies to what I hear from journalists. But it makes a cacophony of sickness... there are many colleagues who are against the vaccine because they have heard everything and anything » *(F2, porter)*<br>« I get angry in front of the TV (...) because some of what they are saying is wrong! (...) It is not always very credible information (...) One has the impression that they have a bone there, and then that they must gnaw at it until it's finished» *(E1, administrative staff)*<br>« They play on people's fears (...) I think it's really the anti-vaccines that are back in the limelight » *(E3, administrative staff)*

	« There is everything and anything that is spread on social networks (...) there are millions of people who have become doctors (in the media)...» *(D3, technical officer)*<br>« If only we could have clearer explanations (...) a little more concrete than what we have at the moment » *(G2, physiotherapist)*

Table S3. Brief timeline of the evolution of COVID-19 vaccination campaign and semi-structured interviews period, at the University Hospital of Bordeaux, from December 2020 to April 2021, France

Dates	Event
**December 2020**	
12.21.20	Pfizer's vaccine authorization in France
12.27.20	Launch of the vaccination campaign<br>- for residents and staff in nursing home,<br>- for people with vulnerable disabilities<br>- for health professionals, professionals working in health or medico-social establishments, aged 50 years old and over and/or suffering from co-morbidity(ies).
**January 2021**	
01.03.21	Moderna's vaccine authorization in France
01.07.21	Interview with A1
01.14.21	Interview with B1
01.18.21	Expansion of the vaccination campaign to include people aged 75 year old and over
01.22.21	Interview with D1 and E1
01.25.21	Interview with C1, E2 and E3
01.26.21	Interview with A2 and D2
**February 2021**	
02.02.21	AstraZeneca's vaccine authorization in France<br>Interview with A3 and B2
02.05.21	Interview with F1 and D3
02.06.21	Extension of vaccination to all health and medico-social professionals, regardless of age
02.15.21	Interview with G1
02.16.21	Interview with G2
02.17.21	Interview with A4 and F2
02.18.21	Interview with B3 and H1
02.19.21	Expansion to the general population between 50 and 64 years old with comorbidities<br>Interview with B4 and C2
**March 2021**	
03.05.21	Interview with C3 and C4

03.12.21	Janssen's vaccine authorization in France
03.15.21	Stop vaccination with AstraZeneca vaccine until further notice from the European Commission
03.19.21	Change of target population with AstraZeneca's vaccine after the reafirmation of the safety and efficacy of the vaccine. AstraZeneca's vaccine its use only for people aged 55 years old and over
03.27.21	Opening to the population aged 70 years old and over
**April 2021**	
04.12.21	Opening to the population over 55 years old, with vaccination in town medicine, in pharmacies or at home
04.16.21	Opening to the population aged 60 years old and over
04.17.21	Interview with A5 and A6
